# (*Z*)-7-[2-(4-Bromo­phen­yl)hydrazin-1-yl­idene]-6-methyl-3-(pyridin-4-yl)-7*H*-1,2,4-triazolo[3,4-*b*][1,3,4]thia­diazine

**DOI:** 10.1107/S1600536812017412

**Published:** 2012-04-25

**Authors:** Hoong-Kun Fun, Suchada Chantrapromma, Mashooq A. Bhat, Hatem A. Abdel-Aziz

**Affiliations:** a X-ray Crystallography Unit, School of Physics, Universiti Sains Malaysia, 11800 USM, Penang, Malaysia; b Crystal Materials Research Unit, Department of Chemistry, Faculty of Science, Prince of Songkla University, Hat-Yai, Songkhla 90112, Thailand; c Department of Pharmaceutical Chemistry, College of Pharmacy, King Saud University, PO Box 2457, Riyadh 11451, Saudi Arabia

## Abstract

In the asymmetric unit of the title compound, C_16_H_12_BrN_7_S, there are two crystallographically independent mol­ecules with similar conformations. Both mol­ecules are slightly twisted; the central 1,2,4-triazolo[3,4-*b*]-1,3,4-thia­diazine ring system makes dihedral angles of 9.65 (15) and 13.29 (15)° with the pyridine and benzene rings, respectively, in one mol­ecule, whereas the corresponding values in the other mol­ecule are 9.30 (15) and 4.84 (15)°. A weak intra­molecular C—H⋯N inter­action with an *S*(6) ring motif is observed in each mol­ecule. In the crystal, the independent mol­ecules are each linked through N—H⋯N hydrogen bonds and weak C—H⋯N interactions into ribbons along the *c* axis. The ribbons are further linked together by weak C—H⋯N, C—H⋯π and π–π [centroid–centroid distances = 3.572 (2)–3.884 (2) Å] inter­actions.

## Related literature

For bond-length data, see: Allen *et al.* (1987 ▶). For hydrogen-bond motifs, see: Bernstein *et al.* (1995 ▶). For background to and the biological activity of [1,2,4]thia­zolo[3,4-*b*][1,3,4]thia­diazine derivatives, see: Abdel-Aziz *et al.* (2007 ▶); Abdel-Wahab *et al.* (2009 ▶); Dawood *et al.* (2005 ▶); Holla *et al.* (2001 ▶); Janin (2007 ▶); Prasad *et al.* (1998 ▶). For the stability of the temperature controller, see: Cosier & Glazer (1986 ▶).
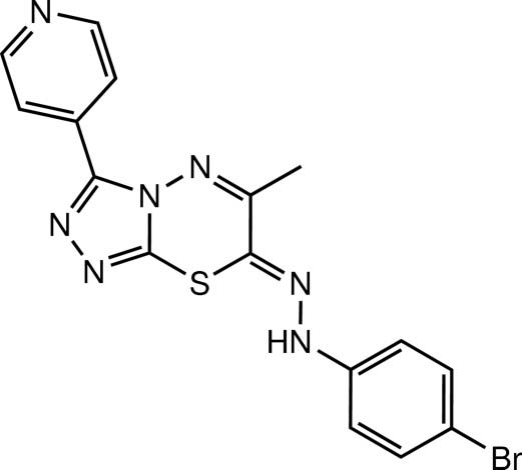


## Experimental

### 

#### Crystal data

C_16_H_12_BrN_7_S
*M* *_r_* = 414.30
Monoclinic, 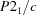

*a* = 17.9868 (7) Å
*b* = 10.3830 (3) Å
*c* = 21.2154 (6) Å
β = 124.671 (2)°
*V* = 3258.6 (2) Å^3^
*Z* = 8
Mo *K*α radiation
μ = 2.67 mm^−1^
*T* = 100 K
0.19 × 0.19 × 0.18 mm

#### Data collection

Bruker APEXII CCD area-detector diffractometer
Absorption correction: multi-scan (*SADABS*; Bruker, 2005 ▶) *T*_min_ = 0.629, *T*_max_ = 0.644
30220 measured reflections
9252 independent reflections
4788 reflections with *I* > 2σ(*I*)
*R*_int_ = 0.058

#### Refinement

*R*[*F* ^2^ > 2σ(*F* ^2^)] = 0.047
*wR*(*F* ^2^) = 0.112
*S* = 0.99
9252 reflections
453 parameters
H-atom parameters constrained
Δρ_max_ = 0.58 e Å^−3^
Δρ_min_ = −0.87 e Å^−3^

### 

Data collection: *APEX2* (Bruker, 2005 ▶); cell refinement: *SAINT* (Bruker, 2005 ▶); data reduction: *SAINT*; program(s) used to solve structure: *SHELXTL* (Sheldrick, 2008 ▶); program(s) used to refine structure: *SHELXTL*; molecular graphics: *SHELXTL*; software used to prepare material for publication: *SHELXTL* (and *PLATON* (Spek, 2009 ▶).

## Supplementary Material

Crystal structure: contains datablock(s) global, I. DOI: 10.1107/S1600536812017412/is5121sup1.cif

Structure factors: contains datablock(s) I. DOI: 10.1107/S1600536812017412/is5121Isup2.hkl

Supplementary material file. DOI: 10.1107/S1600536812017412/is5121Isup3.cml

Additional supplementary materials:  crystallographic information; 3D view; checkCIF report

## Figures and Tables

**Table 1:** Hydrogen-bond geometry (Å, °) *Cg*1 is the centroid of the C6*A*/N4*A*/C7*A*/N5*A*/N6*A* ring.

*D*—H⋯*A*	*D*—H	H⋯*A*	*D*⋯*A*	*D*—H⋯*A*
N1*A*—H*N*1*A*⋯N7*A*^i^	0.82	2.18	2.979 (4)	165
N1*B*—H*N*1*B*⋯N7*B*^i^	0.88	2.15	3.015 (4)	167
C1*A*—H1*AA*⋯N3*A*	0.95	2.40	3.022 (5)	123
C2*A*—H2*AA*⋯N5*B*^ii^	0.95	2.61	3.512 (4)	159
C4*A*—H4*AA*⋯N6*B*^iii^	0.95	2.46	3.257 (5)	141
C1*B*—H1*BA*⋯N3*B*	0.95	2.39	3.023 (5)	123
C4*B*—H4*BA*⋯N6*A*^iii^	0.95	2.41	3.210 (5)	142
C16*A*—H16*B*⋯N3*B*	0.98	2.57	3.445 (5)	149
C16*A*—H16*C*⋯*Cg*1^iv^	0.98	2.72	3.469 (4)	133

Symmetry codes: (i) 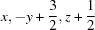; (ii) 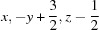; (iii) 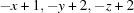; (iv) 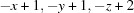.

**Fig. 1.**
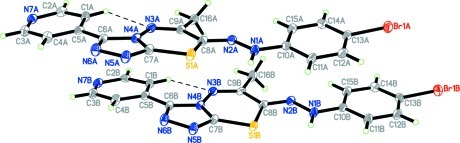
The asymmetric unit of the title compound, showing 40% probability displacement ellipsoids and the atom-numbering scheme. Hydrogen bonds are drawn as dash lines.

**Fig. 2.**
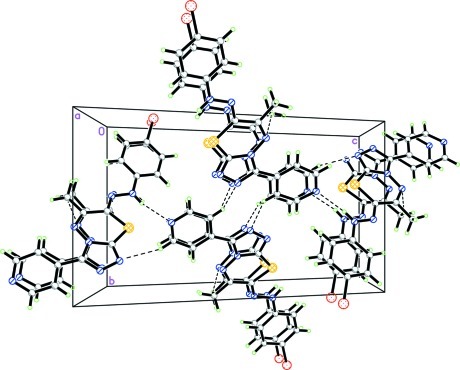
A crystal packing diagram of the title compound viewed along the a axis. Hydrogen bonds are shown as dashed lines.

## supplementary crystallographic information

### Comment

One of the most effective first-line anti-TB drugs is isoniazid (INH). Many analogues featuring the structure of INH have been synthesized and tested as antimycobacterials (Janin, 2007). On the other hand, 1,2,4-triazolo[3,4-*b*]-1,3,4-thiadiazine derivatives have been well documented (Holla *et al.*, 2001; Prasad *et al.*, 1998). In continuation of our interests in the chemistry of the analogs of the title compound (Abdel-Aziz *et al.*, 2007; Abdel-Wahab *et al.*, 2009; Dawood *et al.*, 2005), we reported the synthesis and crystal structure of the title compound (I).

In Fig. 1, there are two crystallographic independent molecules *A* and *B* in the asymmetric unit of (I) with differences in bond angles. The molecule of (I), C_16_H_12_BrN_7_S, is slightly twisted. The middle 1,2,4-triazolo[3,4-*b*]-1,3,4-thiadiazine ring system (C6–C9/N3–N6/S1) makes the dihedral angles of 9.65 (15) and 13.29 (15)° with the pyridyl and benzene rings, respectively in molecule *A* whereas these values are 9.30 (15) and 4.84 (15)° in molecule *B*. Atoms of the 4-bromophenyl-hydrazono fragment (C10–C15/N1/N2/Br1) lie on the same plane with an *r.m.s*. deviation of 0.0270 (1) Å for molecule *A* and 0.0176 (1) Å for molecule *B*. In each molecule a weak intramolecular C—H···N interaction (Fig.1 and Table 1) generates an S(6) ring motif (Bernstein *et al.*, 1995). The bond distances agree with the literature values (Allen *et al.*, 1987).

In the crystal packing (Fig. 2), the molecules are linked into ribbons along the *c* axis by N—H···N hydrogen bonds together with weak C—H···N interactions (Table 1). These ribbons are further stacked along the *a* axis by π–π interactions with the distances of *Cg*2···*Cg*5 = 3.572 (2) Å, *Cg*3···*Cg*4^v^ = 3.884 (2) Å and *Cg*6···*Cg*7^iv^ = 3.617 (2) Å [symmetry code (v) = 1-x, 2-y, 2-z]; *Cg*2, *Cg*3, *Cg*4, *Cg*5, *Cg*6 and *Cg*7 are the centroids of C7A–C9A/N3A/N4A/S1A, C1A–C5A/N7A, C10A–C15A, C6B–C7B/N4B–N6B, C7B–C9B/N3B/N4B/S1B and C10B–C15B rings, respectively. A C—H···π interaction was presented (see Table 1).

### Experimental

To a mixture of 4-amino-5-(pyridin-4-yl)-4*H*-1,2,4-triazole-3-thiol (0.49 g, 2 mmol) and (*Z*)-*N*'-(4-bromophenyl)-2-oxopropanehydrazonoyl chloride (0.55 g, 2 mmol) in ethanol (30 mL), triethylamine (0.2 mL, 2 mmol) was added. The reaction mixture was heated under reflux for 3 h, then left to cool. The precipitated solid was collected by filtration, washed with ethanol, and dried. Golden block-shaped single crystals of the title compound suitable for *X*-ray structure determination were recrystallized from ethanol by slow evaporation of the solvent at room temperature after several days.

### Refinement

All H atoms were placed in calculated positions with *d*(C—H) = 0.95 Å for aromatic and 0.98 Å CH_3_ atoms. The *U*_iso_(H) values were constrained to be 1.5*U*_eq_ of the carrier atom for methyl H atoms and 1.2*U*_eq_ for the remaining H atoms. A rotating group model was used for the methyl groups.

### Crystal data

**Table:**

C_16_H_12_BrN_7_S	*F*(000) = 1664
*M**_r_* = 414.30	*D*_x_ = 1.689 Mg m^−^^3^
Monoclinic, *P*2_1_/*c*	Mo *K*α radiation, λ = 0.71073 Å
Hall symbol: -P 2ybc	Cell parameters from 9252 reflections
*a* = 17.9868 (7) Å	θ = 1.9–30.0°
*b* = 10.3830 (3) Å	µ = 2.67 mm^−^^1^
*c* = 21.2154 (6) Å	*T* = 100 K
β = 124.671 (2)°	Block, gold
*V* = 3258.6 (2) Å^3^	0.19 × 0.19 × 0.18 mm
*Z* = 8	

### Data collection

**Table:**

Bruker APEXII CCD area-detector diffractometer	9252 independent reflections
Radiation source: sealed tube	4788 reflections with *I* > 2σ(*I*)
Graphite monochromator	*R*_int_ = 0.058
φ and ω scans	θ_max_ = 30.0°, θ_min_ = 1.9°
Absorption correction: multi-scan (*SADABS*; Bruker, 2005)	*h* = −24→16
*T*_min_ = 0.629, *T*_max_ = 0.644	*k* = −14→14
30220 measured reflections	*l* = −29→29

### Refinement

**Table:**

Refinement on *F*^2^	Primary atom site location: structure-invariant direct methods
Least-squares matrix: full	Secondary atom site location: difference Fourier map
*R*[*F*^2^ > 2σ(*F*^2^)] = 0.047	Hydrogen site location: inferred from neighbouring sites
*wR*(*F*^2^) = 0.112	H-atom parameters constrained
*S* = 0.99	*w* = 1/[σ^2^(*F*_o_^2^) + (0.0435*P*)^2^] where *P* = (*F*_o_^2^ + 2*F*_c_^2^)/3
9252 reflections	(Δ/σ)_max_ = 0.002
453 parameters	Δρ_max_ = 0.58 e Å^−^^3^
0 restraints	Δρ_min_ = −0.87 e Å^−^^3^

### Special details

**Table:**

Experimental. The crystal was placed in the cold stream of an Oxford Cryosystems Cobra open-flow nitrogen cryostat (Cosier & Glazer, 1986) operating at 120.0 (1) K.
Geometry. All esds (except the esd in the dihedral angle between two l.s. planes) are estimated using the full covariance matrix. The cell esds are taken into account individually in the estimation of esds in distances, angles and torsion angles; correlations between esds in cell parameters are only used when they are defined by crystal symmetry. An approximate (isotropic) treatment of cell esds is used for estimating esds involving l.s. planes.
Refinement. Refinement of F^2^ against ALL reflections. The weighted R-factor wR and goodness of fit S are based on F^2^, conventional R-factors R are based on F, with F set to zero for negative F^2^. The threshold expression of F^2^ > 2sigma(F^2^) is used only for calculating R-factors(gt) etc. and is not relevant to the choice of reflections for refinement. R-factors based on F^2^ are statistically about twice as large as those based on F, and R- factors based on ALL data will be even larger.

### Fractional atomic coordinates and isotropic or equivalent isotropic displacement parameters (Å^2^)

**Table:**

	*x*	*y*	*z*	*U*_iso_*/*U*_eq_	
Br1A	0.21285 (2)	−0.04237 (3)	1.17919 (2)	0.02953 (11)	
S1A	0.47832 (6)	0.62207 (8)	1.11464 (5)	0.0252 (2)	
N1A	0.36955 (18)	0.4220 (2)	1.11905 (15)	0.0209 (6)	
HN1A	0.3936	0.4800	1.1511	0.025*	
N2A	0.35167 (19)	0.4379 (2)	1.04891 (15)	0.0213 (6)	
N3A	0.40347 (18)	0.6140 (3)	0.93433 (15)	0.0211 (6)	
N4A	0.46926 (18)	0.7005 (3)	0.98702 (14)	0.0200 (6)	
N5A	0.56814 (19)	0.8050 (3)	1.09318 (15)	0.0254 (7)	
N6A	0.56960 (19)	0.8564 (3)	1.03296 (15)	0.0262 (7)	
N7A	0.46123 (19)	0.9059 (3)	0.75564 (15)	0.0246 (7)	
C1A	0.4377 (2)	0.7531 (3)	0.82800 (18)	0.0239 (8)	
H1AA	0.4095	0.6761	0.8284	0.029*	
C2A	0.4246 (2)	0.7974 (3)	0.76041 (18)	0.0255 (8)	
H2AA	0.3875	0.7478	0.7152	0.031*	
C3A	0.5160 (2)	0.9708 (3)	0.82084 (19)	0.0256 (8)	
H3AB	0.5447	1.0462	0.8191	0.031*	
C4A	0.5333 (2)	0.9342 (3)	0.89063 (19)	0.0252 (8)	
H4AA	0.5725	0.9842	0.9352	0.030*	
C5A	0.4928 (2)	0.8238 (3)	0.89486 (18)	0.0211 (8)	
C6A	0.5110 (2)	0.7934 (3)	0.97021 (18)	0.0192 (7)	
C7A	0.5078 (2)	0.7137 (3)	1.06399 (18)	0.0214 (8)	
C8A	0.3948 (2)	0.5257 (3)	1.03816 (19)	0.0223 (8)	
C9A	0.3703 (2)	0.5347 (3)	0.95940 (18)	0.0195 (7)	
C10A	0.3285 (2)	0.3169 (3)	1.13031 (18)	0.0197 (7)	
C11A	0.3397 (2)	0.3044 (3)	1.20077 (18)	0.0228 (8)	
H11A	0.3716	0.3685	1.2391	0.027*	
C12A	0.3042 (2)	0.1983 (3)	1.21474 (19)	0.0250 (8)	
H12A	0.3118	0.1893	1.2627	0.030*	
C13A	0.2580 (2)	0.1064 (3)	1.15898 (19)	0.0221 (8)	
C14A	0.2448 (2)	0.1186 (3)	1.08789 (18)	0.0241 (8)	
H14A	0.2121	0.0547	1.0496	0.029*	
C15A	0.2797 (2)	0.2241 (3)	1.07330 (18)	0.0231 (8)	
H15A	0.2705	0.2336	1.0248	0.028*	
C16A	0.2991 (2)	0.4455 (3)	0.89978 (18)	0.0251 (8)	
H16A	0.2913	0.4608	0.8507	0.038*	
H16B	0.2419	0.4615	0.8940	0.038*	
H16C	0.3178	0.3561	0.9157	0.038*	
Br1B	−0.05294 (3)	−0.04795 (4)	1.15572 (2)	0.03264 (11)	
S1B	0.20433 (6)	0.63491 (8)	1.09173 (4)	0.0215 (2)	
N1B	0.11302 (18)	0.4113 (3)	1.10112 (15)	0.0224 (7)	
HN1B	0.1418	0.4704	1.1370	0.027*	
N2B	0.09887 (18)	0.4247 (2)	1.03202 (15)	0.0206 (6)	
N3B	0.14408 (18)	0.6097 (3)	0.91571 (14)	0.0204 (6)	
N4B	0.20336 (17)	0.7039 (2)	0.96629 (13)	0.0175 (6)	
N5B	0.2957 (2)	0.8157 (3)	1.07072 (15)	0.0266 (7)	
N6B	0.30106 (19)	0.8623 (3)	1.01197 (15)	0.0256 (7)	
N7B	0.19972 (19)	0.9085 (3)	0.73658 (15)	0.0246 (7)	
C1B	0.1683 (2)	0.7606 (3)	0.80669 (17)	0.0213 (8)	
H1BA	0.1353	0.6880	0.8055	0.026*	
C2B	0.1566 (2)	0.8074 (3)	0.74018 (18)	0.0228 (8)	
H2BA	0.1144	0.7640	0.6936	0.027*	
C3B	0.2610 (2)	0.9640 (3)	0.80338 (19)	0.0255 (8)	
H3BB	0.2944	1.0345	0.8029	0.031*	
C4B	0.2795 (2)	0.9255 (3)	0.87374 (18)	0.0248 (8)	
H4BA	0.3248	0.9676	0.9197	0.030*	
C5B	0.2301 (2)	0.8236 (3)	0.87509 (18)	0.0200 (8)	
C6B	0.2458 (2)	0.7942 (3)	0.94951 (18)	0.0203 (8)	
C7B	0.2370 (2)	0.7218 (3)	1.04183 (18)	0.0203 (7)	
C8B	0.1359 (2)	0.5194 (3)	1.01993 (17)	0.0197 (8)	
C9B	0.1158 (2)	0.5258 (3)	0.94249 (18)	0.0202 (8)	
C10B	0.0730 (2)	0.3056 (3)	1.11196 (18)	0.0203 (8)	
C11B	0.0878 (2)	0.2884 (3)	1.18328 (18)	0.0266 (8)	
H11B	0.1244	0.3477	1.2235	0.032*	
C12B	0.0489 (2)	0.1838 (3)	1.19575 (19)	0.0281 (9)	
H12B	0.0590	0.1715	1.2444	0.034*	
C13B	−0.0038 (2)	0.0990 (3)	1.1373 (2)	0.0247 (8)	
C14B	−0.0197 (2)	0.1152 (3)	1.06614 (19)	0.0250 (8)	
H14B	−0.0570	0.0560	1.0261	0.030*	
C15B	0.0189 (2)	0.2178 (3)	1.05332 (19)	0.0242 (8)	
H15B	0.0087	0.2286	1.0045	0.029*	
C16B	0.0553 (2)	0.4243 (3)	0.88683 (18)	0.0285 (9)	
H16D	0.0413	0.4447	0.8360	0.043*	
H16E	−0.0008	0.4209	0.8843	0.043*	
H16F	0.0858	0.3406	0.9037	0.043*	

### Atomic displacement parameters (Å^2^)

**Table:**

	*U*^11^	*U*^22^	*U*^33^	*U*^12^	*U*^13^	*U*^23^
Br1A	0.0337 (2)	0.0289 (2)	0.0316 (2)	−0.00298 (18)	0.02195 (19)	0.00247 (17)
S1A	0.0290 (5)	0.0318 (5)	0.0180 (4)	−0.0060 (4)	0.0152 (4)	−0.0021 (4)
N1A	0.0248 (17)	0.0234 (15)	0.0168 (14)	−0.0031 (13)	0.0133 (13)	−0.0014 (11)
N2A	0.0245 (17)	0.0243 (16)	0.0159 (14)	0.0023 (13)	0.0119 (13)	0.0016 (12)
N3A	0.0201 (16)	0.0252 (16)	0.0195 (15)	−0.0024 (14)	0.0122 (13)	−0.0040 (13)
N4A	0.0198 (16)	0.0267 (15)	0.0144 (14)	0.0002 (13)	0.0103 (13)	−0.0007 (12)
N5A	0.0294 (18)	0.0316 (17)	0.0175 (15)	−0.0040 (15)	0.0148 (14)	0.0003 (13)
N6A	0.0287 (18)	0.0312 (17)	0.0186 (15)	−0.0044 (14)	0.0134 (15)	0.0008 (13)
N7A	0.0254 (18)	0.0296 (16)	0.0210 (15)	0.0036 (14)	0.0146 (14)	0.0035 (13)
C1A	0.026 (2)	0.0269 (19)	0.0203 (18)	−0.0006 (16)	0.0140 (17)	0.0012 (15)
C2A	0.020 (2)	0.035 (2)	0.0160 (18)	0.0024 (18)	0.0072 (16)	−0.0011 (16)
C3A	0.026 (2)	0.029 (2)	0.0249 (19)	−0.0016 (17)	0.0158 (17)	0.0027 (16)
C4A	0.0192 (19)	0.031 (2)	0.0198 (18)	−0.0011 (16)	0.0079 (16)	0.0012 (15)
C5A	0.0165 (19)	0.0266 (19)	0.0212 (18)	0.0062 (16)	0.0113 (16)	0.0027 (15)
C6A	0.0160 (19)	0.0242 (18)	0.0167 (17)	0.0004 (15)	0.0088 (16)	0.0000 (15)
C7A	0.022 (2)	0.0267 (18)	0.0158 (17)	0.0005 (16)	0.0107 (16)	0.0017 (15)
C8A	0.0216 (19)	0.028 (2)	0.0178 (17)	0.0022 (17)	0.0115 (16)	0.0008 (15)
C9A	0.0172 (18)	0.0246 (18)	0.0169 (17)	0.0029 (16)	0.0098 (15)	0.0011 (15)
C10A	0.0158 (18)	0.0219 (17)	0.0209 (17)	0.0024 (15)	0.0102 (16)	0.0031 (14)
C11A	0.0205 (19)	0.0308 (19)	0.0181 (17)	−0.0032 (16)	0.0116 (16)	−0.0019 (15)
C12A	0.025 (2)	0.031 (2)	0.0224 (19)	0.0028 (17)	0.0152 (17)	0.0016 (16)
C13A	0.020 (2)	0.0263 (18)	0.0213 (18)	0.0025 (16)	0.0127 (16)	0.0028 (15)
C14A	0.024 (2)	0.0242 (19)	0.0214 (18)	−0.0004 (17)	0.0110 (17)	−0.0014 (15)
C15A	0.0193 (19)	0.031 (2)	0.0185 (18)	0.0030 (17)	0.0102 (16)	0.0034 (16)
C16A	0.027 (2)	0.0300 (19)	0.0217 (18)	−0.0035 (17)	0.0162 (17)	−0.0045 (16)
Br1B	0.0373 (2)	0.0280 (2)	0.0405 (2)	−0.00200 (19)	0.0268 (2)	0.00421 (18)
S1B	0.0253 (5)	0.0267 (5)	0.0144 (4)	−0.0035 (4)	0.0124 (4)	−0.0015 (4)
N1B	0.0283 (18)	0.0259 (15)	0.0159 (15)	−0.0047 (14)	0.0143 (14)	−0.0020 (12)
N2B	0.0185 (16)	0.0257 (16)	0.0177 (15)	0.0020 (13)	0.0103 (13)	0.0016 (12)
N3B	0.0212 (16)	0.0213 (15)	0.0192 (15)	−0.0031 (13)	0.0117 (14)	−0.0046 (12)
N4B	0.0174 (15)	0.0235 (15)	0.0113 (13)	−0.0003 (13)	0.0079 (13)	0.0000 (11)
N5B	0.0315 (18)	0.0340 (17)	0.0162 (15)	−0.0068 (15)	0.0146 (14)	−0.0012 (13)
N6B	0.0284 (18)	0.0310 (16)	0.0185 (15)	−0.0072 (14)	0.0140 (14)	−0.0008 (13)
N7B	0.0292 (18)	0.0309 (16)	0.0171 (15)	0.0028 (14)	0.0151 (15)	0.0016 (13)
C1B	0.023 (2)	0.0227 (18)	0.0177 (17)	0.0019 (16)	0.0116 (16)	0.0027 (14)
C2B	0.026 (2)	0.0247 (19)	0.0165 (17)	0.0066 (17)	0.0110 (17)	0.0002 (15)
C3B	0.028 (2)	0.0287 (19)	0.0247 (19)	0.0005 (18)	0.0180 (17)	0.0041 (16)
C4B	0.022 (2)	0.034 (2)	0.0142 (17)	−0.0019 (17)	0.0085 (16)	0.0015 (15)
C5B	0.0199 (19)	0.0259 (18)	0.0144 (17)	0.0042 (16)	0.0098 (16)	0.0042 (14)
C6B	0.021 (2)	0.0224 (18)	0.0178 (17)	−0.0001 (16)	0.0112 (16)	0.0013 (15)
C7B	0.0190 (19)	0.0259 (18)	0.0167 (17)	−0.0007 (16)	0.0105 (16)	0.0010 (15)
C8B	0.0182 (18)	0.0262 (19)	0.0155 (17)	0.0019 (16)	0.0102 (15)	−0.0010 (15)
C9B	0.0198 (19)	0.0256 (19)	0.0181 (17)	0.0005 (16)	0.0125 (16)	−0.0033 (15)
C10B	0.022 (2)	0.0219 (18)	0.0221 (18)	−0.0018 (16)	0.0153 (17)	−0.0008 (15)
C11B	0.034 (2)	0.0278 (19)	0.0216 (19)	−0.0062 (18)	0.0175 (18)	−0.0042 (15)
C12B	0.033 (2)	0.034 (2)	0.0225 (19)	0.0019 (18)	0.0188 (18)	0.0031 (16)
C13B	0.022 (2)	0.0236 (18)	0.031 (2)	0.0010 (17)	0.0168 (18)	0.0042 (16)
C14B	0.022 (2)	0.0259 (19)	0.0236 (19)	−0.0023 (17)	0.0110 (17)	−0.0021 (16)
C15B	0.023 (2)	0.032 (2)	0.0195 (18)	0.0016 (17)	0.0127 (17)	0.0017 (16)
C16B	0.033 (2)	0.034 (2)	0.0200 (18)	−0.0089 (18)	0.0166 (18)	−0.0064 (15)

### Geometric parameters (Å, º)

**Table:**

Br1A—C13A	1.904 (3)	Br1B—C13B	1.912 (3)
S1A—C7A	1.728 (3)	S1B—C7B	1.728 (3)
S1A—C8A	1.766 (4)	S1B—C8B	1.772 (3)
N1A—N2A	1.342 (3)	N1B—N2B	1.345 (3)
N1A—C10A	1.413 (4)	N1B—C10B	1.401 (4)
N1A—HN1A	0.8231	N1B—HN1B	0.8809
N2A—C8A	1.298 (4)	N2B—C8B	1.293 (4)
N3A—C9A	1.295 (4)	N3B—C9B	1.292 (4)
N3A—N4A	1.397 (3)	N3B—N4B	1.394 (3)
N4A—C7A	1.371 (4)	N4B—C7B	1.366 (4)
N4A—C6A	1.388 (4)	N4B—C6B	1.377 (4)
N5A—C7A	1.303 (4)	N5B—C7B	1.306 (4)
N5A—N6A	1.398 (3)	N5B—N6B	1.391 (3)
N6A—C6A	1.309 (4)	N6B—C6B	1.322 (4)
N7A—C3A	1.337 (4)	N7B—C3B	1.333 (4)
N7A—C2A	1.338 (4)	N7B—C2B	1.333 (4)
C1A—C5A	1.391 (4)	C1B—C2B	1.390 (4)
C1A—C2A	1.392 (4)	C1B—C5B	1.391 (4)
C1A—H1AA	0.9500	C1B—H1BA	0.9500
C2A—H2AA	0.9500	C2B—H2BA	0.9500
C3A—C4A	1.381 (4)	C3B—C4B	1.391 (4)
C3A—H3AB	0.9500	C3B—H3BB	0.9500
C4A—C5A	1.388 (4)	C4B—C5B	1.393 (4)
C4A—H4AA	0.9500	C4B—H4BA	0.9500
C5A—C6A	1.472 (4)	C5B—C6B	1.469 (4)
C8A—C9A	1.469 (4)	C8B—C9B	1.469 (4)
C9A—C16A	1.501 (4)	C9B—C16B	1.493 (4)
C10A—C11A	1.396 (4)	C10B—C11B	1.391 (4)
C10A—C15A	1.397 (4)	C10B—C15B	1.396 (4)
C11A—C12A	1.387 (4)	C11B—C12B	1.396 (4)
C11A—H11A	0.9500	C11B—H11B	0.9500
C12A—C13A	1.371 (4)	C12B—C13B	1.368 (5)
C12A—H12A	0.9500	C12B—H12B	0.9500
C13A—C14A	1.393 (4)	C13B—C14B	1.378 (4)
C14A—C15A	1.382 (4)	C14B—C15B	1.380 (4)
C14A—H14A	0.9500	C14B—H14B	0.9500
C15A—H15A	0.9500	C15B—H15B	0.9500
C16A—H16A	0.9800	C16B—H16D	0.9800
C16A—H16B	0.9800	C16B—H16E	0.9800
C16A—H16C	0.9800	C16B—H16F	0.9800
			
C7A—S1A—C8A	98.18 (16)	C7B—S1B—C8B	97.95 (16)
N2A—N1A—C10A	117.7 (3)	N2B—N1B—C10B	117.6 (3)
N2A—N1A—HN1A	120.6	N2B—N1B—HN1B	121.6
C10A—N1A—HN1A	120.2	C10B—N1B—HN1B	120.5
C8A—N2A—N1A	119.4 (3)	C8B—N2B—N1B	119.7 (3)
C9A—N3A—N4A	117.3 (3)	C9B—N3B—N4B	117.0 (3)
C7A—N4A—C6A	104.8 (3)	C7B—N4B—C6B	105.7 (3)
C7A—N4A—N3A	129.9 (3)	C7B—N4B—N3B	129.3 (3)
C6A—N4A—N3A	125.2 (3)	C6B—N4B—N3B	124.9 (2)
C7A—N5A—N6A	106.7 (3)	C7B—N5B—N6B	106.8 (3)
C6A—N6A—N5A	108.4 (3)	C6B—N6B—N5B	108.4 (3)
C3A—N7A—C2A	116.8 (3)	C3B—N7B—C2B	115.8 (3)
C5A—C1A—C2A	118.7 (3)	C2B—C1B—C5B	117.7 (3)
C5A—C1A—H1AA	120.6	C2B—C1B—H1BA	121.2
C2A—C1A—H1AA	120.6	C5B—C1B—H1BA	121.2
N7A—C2A—C1A	123.6 (3)	N7B—C2B—C1B	125.2 (3)
N7A—C2A—H2AA	118.2	N7B—C2B—H2BA	117.4
C1A—C2A—H2AA	118.2	C1B—C2B—H2BA	117.4
N7A—C3A—C4A	123.7 (3)	N7B—C3B—C4B	124.4 (3)
N7A—C3A—H3AB	118.2	N7B—C3B—H3BB	117.8
C4A—C3A—H3AB	118.2	C4B—C3B—H3BB	117.8
C3A—C4A—C5A	119.3 (3)	C3B—C4B—C5B	118.3 (3)
C3A—C4A—H4AA	120.4	C3B—C4B—H4BA	120.8
C5A—C4A—H4AA	120.4	C5B—C4B—H4BA	120.8
C4A—C5A—C1A	117.8 (3)	C1B—C5B—C4B	118.4 (3)
C4A—C5A—C6A	116.6 (3)	C1B—C5B—C6B	125.1 (3)
C1A—C5A—C6A	125.7 (3)	C4B—C5B—C6B	116.5 (3)
N6A—C6A—N4A	109.0 (3)	N6B—C6B—N4B	108.5 (3)
N6A—C6A—C5A	123.3 (3)	N6B—C6B—C5B	122.8 (3)
N4A—C6A—C5A	127.6 (3)	N4B—C6B—C5B	128.6 (3)
N5A—C7A—N4A	111.0 (3)	N5B—C7B—N4B	110.6 (3)
N5A—C7A—S1A	124.9 (2)	N5B—C7B—S1B	124.6 (2)
N4A—C7A—S1A	124.0 (3)	N4B—C7B—S1B	124.8 (3)
N2A—C8A—C9A	115.0 (3)	N2B—C8B—C9B	115.4 (3)
N2A—C8A—S1A	121.2 (3)	N2B—C8B—S1B	121.9 (2)
C9A—C8A—S1A	123.8 (3)	C9B—C8B—S1B	122.7 (3)
N3A—C9A—C8A	126.7 (3)	N3B—C9B—C8B	127.9 (3)
N3A—C9A—C16A	114.7 (3)	N3B—C9B—C16B	114.9 (3)
C8A—C9A—C16A	118.6 (3)	C8B—C9B—C16B	117.2 (3)
C11A—C10A—C15A	120.0 (3)	C11B—C10B—C15B	119.4 (3)
C11A—C10A—N1A	118.8 (3)	C11B—C10B—N1B	118.8 (3)
C15A—C10A—N1A	121.2 (3)	C15B—C10B—N1B	121.9 (3)
C12A—C11A—C10A	119.9 (3)	C10B—C11B—C12B	120.0 (3)
C12A—C11A—H11A	120.0	C10B—C11B—H11B	120.0
C10A—C11A—H11A	120.0	C12B—C11B—H11B	120.0
C13A—C12A—C11A	119.6 (3)	C13B—C12B—C11B	119.5 (3)
C13A—C12A—H12A	120.2	C13B—C12B—H12B	120.3
C11A—C12A—H12A	120.2	C11B—C12B—H12B	120.3
C12A—C13A—C14A	121.2 (3)	C12B—C13B—C14B	121.3 (3)
C12A—C13A—Br1A	119.8 (3)	C12B—C13B—Br1B	119.4 (3)
C14A—C13A—Br1A	119.1 (3)	C14B—C13B—Br1B	119.3 (3)
C15A—C14A—C13A	119.7 (3)	C13B—C14B—C15B	119.7 (3)
C15A—C14A—H14A	120.2	C13B—C14B—H14B	120.1
C13A—C14A—H14A	120.2	C15B—C14B—H14B	120.1
C14A—C15A—C10A	119.6 (3)	C14B—C15B—C10B	120.1 (3)
C14A—C15A—H15A	120.2	C14B—C15B—H15B	119.9
C10A—C15A—H15A	120.2	C10B—C15B—H15B	119.9
C9A—C16A—H16A	109.5	C9B—C16B—H16D	109.5
C9A—C16A—H16B	109.5	C9B—C16B—H16E	109.5
H16A—C16A—H16B	109.5	H16D—C16B—H16E	109.5
C9A—C16A—H16C	109.5	C9B—C16B—H16F	109.5
H16A—C16A—H16C	109.5	H16D—C16B—H16F	109.5
H16B—C16A—H16C	109.5	H16E—C16B—H16F	109.5
			
C10A—N1A—N2A—C8A	−173.4 (3)	C10B—N1B—N2B—C8B	−178.7 (3)
C9A—N3A—N4A—C7A	2.0 (5)	C9B—N3B—N4B—C7B	−0.1 (5)
C9A—N3A—N4A—C6A	−178.9 (3)	C9B—N3B—N4B—C6B	174.5 (3)
C7A—N5A—N6A—C6A	−0.7 (4)	C7B—N5B—N6B—C6B	−0.5 (4)
C3A—N7A—C2A—C1A	2.7 (5)	C3B—N7B—C2B—C1B	2.7 (5)
C5A—C1A—C2A—N7A	−0.9 (5)	C5B—C1B—C2B—N7B	−0.2 (5)
C2A—N7A—C3A—C4A	−2.5 (5)	C2B—N7B—C3B—C4B	−2.0 (5)
N7A—C3A—C4A—C5A	0.5 (5)	N7B—C3B—C4B—C5B	−1.1 (5)
C3A—C4A—C5A—C1A	1.4 (5)	C2B—C1B—C5B—C4B	−3.0 (5)
C3A—C4A—C5A—C6A	−177.5 (3)	C2B—C1B—C5B—C6B	174.8 (3)
C2A—C1A—C5A—C4A	−1.2 (5)	C3B—C4B—C5B—C1B	3.6 (5)
C2A—C1A—C5A—C6A	177.6 (3)	C3B—C4B—C5B—C6B	−174.4 (3)
N5A—N6A—C6A—N4A	0.4 (4)	N5B—N6B—C6B—N4B	0.6 (4)
N5A—N6A—C6A—C5A	178.4 (3)	N5B—N6B—C6B—C5B	175.8 (3)
C7A—N4A—C6A—N6A	−0.1 (3)	C7B—N4B—C6B—N6B	−0.4 (4)
N3A—N4A—C6A—N6A	−179.4 (3)	N3B—N4B—C6B—N6B	−176.1 (3)
C7A—N4A—C6A—C5A	−177.9 (3)	C7B—N4B—C6B—C5B	−175.3 (3)
N3A—N4A—C6A—C5A	2.8 (5)	N3B—N4B—C6B—C5B	9.0 (5)
C4A—C5A—C6A—N6A	−7.5 (5)	C1B—C5B—C6B—N6B	−175.5 (3)
C1A—C5A—C6A—N6A	173.8 (3)	C4B—C5B—C6B—N6B	2.3 (5)
C4A—C5A—C6A—N4A	170.1 (3)	C1B—C5B—C6B—N4B	−1.3 (5)
C1A—C5A—C6A—N4A	−8.6 (5)	C4B—C5B—C6B—N4B	176.5 (3)
N6A—N5A—C7A—N4A	0.6 (4)	N6B—N5B—C7B—N4B	0.2 (4)
N6A—N5A—C7A—S1A	−178.5 (2)	N6B—N5B—C7B—S1B	−178.3 (2)
C6A—N4A—C7A—N5A	−0.4 (4)	C6B—N4B—C7B—N5B	0.1 (4)
N3A—N4A—C7A—N5A	178.9 (3)	N3B—N4B—C7B—N5B	175.5 (3)
C6A—N4A—C7A—S1A	178.8 (2)	C6B—N4B—C7B—S1B	178.6 (2)
N3A—N4A—C7A—S1A	−1.9 (5)	N3B—N4B—C7B—S1B	−5.9 (5)
C8A—S1A—C7A—N5A	179.2 (3)	C8B—S1B—C7B—N5B	−174.2 (3)
C8A—S1A—C7A—N4A	0.1 (3)	C8B—S1B—C7B—N4B	7.5 (3)
N1A—N2A—C8A—C9A	179.8 (3)	N1B—N2B—C8B—C9B	−179.7 (3)
N1A—N2A—C8A—S1A	1.4 (4)	N1B—N2B—C8B—S1B	−0.8 (4)
C7A—S1A—C8A—N2A	179.7 (3)	C7B—S1B—C8B—N2B	175.8 (3)
C7A—S1A—C8A—C9A	1.5 (3)	C7B—S1B—C8B—C9B	−5.5 (3)
N4A—N3A—C9A—C8A	0.0 (5)	N4B—N3B—C9B—C8B	2.4 (5)
N4A—N3A—C9A—C16A	179.3 (3)	N4B—N3B—C9B—C16B	−177.8 (3)
N2A—C8A—C9A—N3A	179.9 (3)	N2B—C8B—C9B—N3B	−179.8 (3)
S1A—C8A—C9A—N3A	−1.8 (5)	S1B—C8B—C9B—N3B	1.3 (5)
N2A—C8A—C9A—C16A	0.7 (5)	N2B—C8B—C9B—C16B	0.4 (4)
S1A—C8A—C9A—C16A	179.0 (2)	S1B—C8B—C9B—C16B	−178.5 (2)
N2A—N1A—C10A—C11A	−174.6 (3)	N2B—N1B—C10B—C11B	178.9 (3)
N2A—N1A—C10A—C15A	7.4 (4)	N2B—N1B—C10B—C15B	−1.1 (4)
C15A—C10A—C11A—C12A	1.5 (5)	C15B—C10B—C11B—C12B	0.1 (5)
N1A—C10A—C11A—C12A	−176.5 (3)	N1B—C10B—C11B—C12B	−179.9 (3)
C10A—C11A—C12A—C13A	−0.2 (5)	C10B—C11B—C12B—C13B	−0.1 (5)
C11A—C12A—C13A—C14A	−1.0 (5)	C11B—C12B—C13B—C14B	−0.3 (5)
C11A—C12A—C13A—Br1A	177.8 (2)	C11B—C12B—C13B—Br1B	177.5 (3)
C12A—C13A—C14A—C15A	0.8 (5)	C12B—C13B—C14B—C15B	0.8 (5)
Br1A—C13A—C14A—C15A	−178.0 (2)	Br1B—C13B—C14B—C15B	−177.1 (2)
C13A—C14A—C15A—C10A	0.6 (5)	C13B—C14B—C15B—C10B	−0.7 (5)
C11A—C10A—C15A—C14A	−1.7 (5)	C11B—C10B—C15B—C14B	0.3 (5)
N1A—C10A—C15A—C14A	176.3 (3)	N1B—C10B—C15B—C14B	−179.7 (3)

### Hydrogen-bond geometry (Å, º)

*Cg*1 is the centroid of the C6*A*/N4*A*/C7*A*/N5*A*/N6*A* ring.

**Table:**

*D*—H···*A*	*D*—H	H···*A*	*D*···*A*	*D*—H···*A*
N1*A*—H*N*1*A*···N7*A*^i^	0.82	2.18	2.979 (4)	165
N1*B*—H*N*1*B*···N7*B*^i^	0.88	2.15	3.015 (4)	167
C1*A*—H1*AA*···N3*A*	0.95	2.40	3.022 (5)	123
C2*A*—H2*AA*···N5*B*^ii^	0.95	2.61	3.512 (4)	159
C4*A*—H4*AA*···N6*B*^iii^	0.95	2.46	3.257 (5)	141
C1*B*—H1*BA*···N3*B*	0.95	2.39	3.023 (5)	123
C4*B*—H4*BA*···N6*A*^iii^	0.95	2.41	3.210 (5)	142
C16*A*—H16*B*···N3*B*	0.98	2.57	3.445 (5)	149
C16*A*—H16*C*···*Cg*1^iv^	0.98	2.72	3.469 (4)	133

Symmetry codes: (i) *x*, −*y*+3/2, *z*+1/2; (ii) *x*, −*y*+3/2, *z*−1/2; (iii) −*x*+1, −*y*+2, −*z*+2; (iv) −*x*+1, −*y*+1, −*z*+2.
